# Supplementary material for: Landmark-based spatial navigation across the human lifespan
Source: eLife. 2023 Mar 13;12:e81318. doi: 10.7554/eLife.81318 (PMC10036117; doi:10.7554/eLife.81318)
Supplement: Supplementary file 1. [file elife-81318-supp1.docx]

|  | | **Children** | | **Young adults** | | **Older adults** | |
| --- | --- | --- | --- | --- | --- | --- | --- |
|  |  | **n** | **mean age (std)** | **n** | **mean age (std)** | **n** | **mean age (std)** |
| **Landmark condition** | **ALLO** | 5 | 10.40 (0.55) | 9 | 28.00 (5.35) | 6 | 72.67 (5.43) |
|  | **EGO** | 9 | 10.56 (0.53) | 1 | 25 | 10 | 73.00 (3.16) |
|  | **RETURN** | 1 | 10 | 0 |  | 1 | 76 |
|  | **total** | 15 | 10.46 (0.52) | 10 | 27.7 (5.14) | 17 | 73.06 (3.93) |
| **Geometry condition** | **ALLO** | 12 | 10.33 (0.49) | 12 | 28.08 (3.65) | 9 | 73.33 (4.53) |
|  | **EGO** | 2 | 10.00 (0) | 0 |  | 2 | 72.50 (0.70) |
|  | **RETURN** | 0 |  | 0 |  | 0 |  |
|  | **total** | 14 | 10.29 (0.47) | 12 | 28.08 (3.65) | 11 | 73.18 (4.07) |
