# Supplementary material for: Landmark-based spatial navigation across the human lifespan
Source: eLife. 2023 Mar 13;12:e81318. doi: 10.7554/eLife.81318 (PMC10036117; doi:10.7554/eLife.81318)
Supplement: Supplementary file 2. [file elife-81318-supp2.docx]

|  | | **Young adults** | | **Older adults** | |
| --- | --- | --- | --- | --- | --- |
|  |  | **n** | **mean age (std)** | **n** | **mean age (std)** |
| **Landmark condition**  **-**  **Real-world setting** | **ALLO** | 8 | 28.16 (3.62) | 3 | 72.61 (3.21) |
|  | **EGO** | 1 | 22 | 5 | 72.27 (5.29) |
|  | **RETURN** | 0 |  | 0 |  |
|  | **total** | 9 | 27.39 (4) | 8 | 72.40 (4) |
