# Supplementary material for: Landmark-based spatial navigation across the human lifespan
Source: eLife. 2023 Mar 13;12:e81318. doi: 10.7554/eLife.81318 (PMC10036117; doi:10.7554/eLife.81318)
Supplement: Supplementary file 3. [file elife-81318-supp3.docx]

|  | **Assessed function** | **Test/apparatus, task description and units of measurement** |
| --- | --- | --- |
| **Visual screening** | Contrast sensitivity | Evaluating sensitivity thresholds for different spatial frequencies (0.5, 1, 2, 4, 8, 16 circles per degree) in photopic condition, with subjects’ own optical correction |
|  | Visual attention | Useful Field of View: central (UFOV1), central+peripheral visual discrimination task, without (UFOV2) and with (UFOV3) visual distractors, with subjects’ own optical correction. Expressed in second needed for a correct discrimination (UFOV, Ball & Owsley, 1993) |
| **Cognitive screening** | Composite | Mini Mental State Examination (MMSE, Folstein, Folstein, & McHugh, 1975). Expressed as a score representing items succeeded. |
|  | Spatial working memory | Corsi block-tapping test: recalling spatial sequence of cubes in the forward (short-term span) or backward (working memory span) order (Schuhfried, 2004). Expressed as the largest sequence succeeded. |
|  | Visual memory | Figural Memory test: learning 9 visual figures presented 5 times and recalling them after 5 and 20 minutes (Schuhfried, 2004). Expressed as number of figures learned correctly (sum up to 45 items) or recalled (up to 9 items) |
|  | Mental rotation | 3D mental rotation test: mentally imaging views around 3D cubes (Schuhfried, 2004). Expressed as a number of correct items (up to 30) |
|  | Perspective taking | Perspective Taking/Spatial Orientation test: imagining position and facing direction relative to a two-dimensional array of objects and indicating the position of a third object (Hegarty & Waller, 2004). Expressed as an error in degree |
|  | Mental flexibility | Trail Making test: following sequences of letters (part A) and alternating between letters and numbers (B). Expressed as a difference between time needed in B and A (Schuhfried, 2004) |
|  | Inhibition capacity | Go/no go task: responding to a frequent stimulus while inhibiting response to a rare one (Schuhfried, 2004). Expressed as a sensitivity index: z(hits) – z(false alarm) |
