# Supplementary material for: Landmark-based spatial navigation across the human lifespan
Source: eLife. 2023 Mar 13;12:e81318. doi: 10.7554/eLife.81318 (PMC10036117; doi:10.7554/eLife.81318)
Supplement: Supplementary file 4. [file elife-81318-supp4.docx]

| **Inclusion criteria** | - Volunteer: man or woman - Aged over 18 years old - Affiliated to the social security system - Absence of pathology, deficit or disorder that can interfere with visual, auditory, vestibular or cognitive functions |
| --- | --- |
| **Exclusion criteria** | - Person under guardianship - Person using walker or wheelchair - Person with a history of stroke - Person with a history of epilepsy or convulsions - Person with an history of active or progressive ophtalmological pathology to the exception of cataract - Person with an history of active or progressive otological pathology or a surgical treatment (cholesteatoma, neuroma, otosclerosis) - Best corrected visual acuity at 100% contrast lower than 7/10 or 5/10 before or after 70 yo, respectively, in one or both eyes - Presence of abnormality on the monocular or binocular field of view - Impaired color vision for one or both eyes on the D15 desaturated Lanthony test (protanope, deuteranope or tritanope) - Ascending audiometric curve for one or both ears - Balance disorders - Minimal Mental State exam score lower than 24 - General Health Questionnaire score higher than 4 for depressive items |
